# Higher education and science popularization: Can they achieve coordinated growth?

**DOI:** 10.1371/journal.pone.0256612

**Published:** 2021-09-07

**Authors:** Yuqing Geng, Yan Yan

**Affiliations:** School of Business, Shanghai Dianji University, Shanghai, China; University of Defence in Belgrade, SERBIA

## Abstract

This study aims to explore whether higher education and science popularization can achieve coordinated growth with temporal and spatial characteristics. Selecting the provincial regions of the Yangtze River Economic Belt in China as cases with data from the national statistics administrations (such as China Statistical Yearbook), this study uses entropy weight analysis, TOPSIS, GM(1,1) gray prediction methods and coupling coordination degree model to evaluate the coordinated growth status. The key findings are: (1) the annual budget per student, and the number of science and technology museums affect both systems more obviously; (2) the overall performances of science popularization fluctuate more obviously than those of higher education; (3) the coordinated growth performances of the two systems in most regions remain mild fluctuations and keep relatively stable coordinated status, however, temporal and spatial variation tendencies do exist among regions. Therefore, corresponding countermeasures should be implemented: generally, national authority needs to involve in coordination activities among regions; the regions with satisfactory coordinated growth performances need more creative approaches to maintain the coordinated growth interactions; the regions at the transitioning status need to prevent the grade decline and upgrade the performances; the regions with lagging performances need to stop the decline and reduce the gaps with others. The novelties include analyzing the coordinated growth interaction mechanism between the two, selecting indices to assess the abstract interaction mechanism precisely, proposing suggestions based on temporal and spatial comparisons of the coordinated growth performances, etc.

## Introduction

Higher education and science popularization play important roles in social development and in promoting mutual growth [1, 2]. Higher education in this paper refers to the situation in which the local higher education industry is competitive and interacts with other elements; higher education is able to evolve together with other factors by providing related educational services, and it can also reflect the possible problems of education industry or other industries such as science popularization [3, 4]. Science popularization in this paper refers to the situation in which the public obtain the knowledge, skills, thoughts, methodologies, and values of sciences via various effective approaches [5]. Higher education and science popularization are two different systems; however, they enjoy certain similarities: the both systems are important in promoting social growth; they have the same influential factors such as individual participants and public funds; they both belong to education industry (higher education and social education); and they both can cultivate local residents [6–8]. Therefore, it is needed to evaluate the overall performances of the two systems and to enhance their performances accordingly.

It is important to achieve coordinated growth between higher education and science popularization; however, higher education and science popularization are tied with complexity: there are correlations between them, meanwhile it is still doubtful how they realize interactions and achieve coordinated development. Previous studies show that higher education affects science popularization activities with both positive and negative results. For instance, competitive higher education enhances the effectiveness of science popularization, whereas deficiencies of higher education such as less qualified textbooks or lacking sufficient science education decrease the persuasiveness and conviction of science popularization [9, 10]. Besides, it is also shown that science popularization affects higher education with bi-directional results. For example, science popularization affects university students’ passion for study so that the effectiveness of higher education is enhanced, whereas science popularization occupies the resources of higher education and hinder its further growth [11, 12]. Therefore, it is significant to explore their coordinated growth mechanism and realize their benign interactions.

Currently, it is still less clear how they evolve each other and achieve coordinated growth; considering this problem, we hereby justify our research question: can higher education and science popularization achieve coordinated growth? If so, are there any characteristics of the coordinated growth? The answers to the research question will help us to enhance the coordinated development of the two systems more effectively and efficiently, so that social development can be further realized. To answer this question, it is needed to understand the interactive coordination mechanism between higher education and science popularization, confirm the main influential factors, evaluate how they achieve benign coordination, and even forecast the tendency of the coordinated growth so that we can take corresponding measures to improve the coordinated growth between higher education and science popularization in the future.

In this paper, we firstly constructed the coordination model to explore the coordinated growth relationship between higher education and science popularization, secondly evaluated the main influential factors, the overall performances of the two systems, and the coordinated growth performances between them based on the case of the Yangtze River Economic Belt in China, thirdly forecasted the tendency of the coordinated growth performances in the next years, and fourth proposed suggestions to enhance mutual coordinated growth of both higher education and science popularization.

## Literature review

In this research, a systematic literature review was carefully conducted with the publications regarding the topics of higher education, science popularization, and research methods or models in multi-criteria decision being reviewed. The research papers were mainly indexed in SCI and SSCI, and were published from the year of 2011 to the year of 2021 (mainly in the latest 2–3 years). Besides, these papers used different research methodologies (both qualitatively and quantitatively), guaranteeing the completeness of the literature review.

### Effects: Higher education on science popularization

Higher education has both positive and negative effects on science popularization. From the positive effects’ perspective, better higher education performances usually mean higher educational budgets, better infrastructures support, more effective personnel training programs, and more power in controling media and public resources, which lead to better outcomes of science popularization [13, 14]; cases in certain schools and communities have proved that higher education has accelerated obvious development of science popularization [8]. Besides, the outcomes of higher education include science popularization activities, and higher education activities increases the spread impacts and effectiveness of science popularization; for instance, by organizing various science and technology related competitions, universities promote students’ interests in sciences so that the influence of science popularization activities increases [15, 16]. In addition, some elements of higher education, such as the latest research hotspot in academia, the key persons in research fields, the supportive actions or regulations of universities, etc., are more likely to guide the development direction of science popularization, and to enhance the authoritativeness of science popularization [17–20]. It is also found that the experimental research results in universities can be directly applied in science popularization activities, and then make positive effects [21, 22].

From the negative effects’ perspective, higher education can lead to several problems of science popularization. Specialists in the higher education system are able to suggest or guide which content or information of science popularization should be provided, and they may deceive the public intentionally or unintentionally by providing biased or even wrong information so that the understanding of the public may be misled and the outcomes of science popularization may be unwelcomed [12, 23]. In addition, local higher education system occupies a certain proportion of the limited local resources such as labor resources, financial resources, land resources, etc., which squeezes the resources of science popularization and hinders the growth of science popularization [24]. What is more, some elements of higher education may hinder the attractiveness of science popularization activities; for instance, some academic research articles written by university faculties are unintelligible and obscure, which decreases the enthusiasm of public to science and technology, reduces the effects of science popularization activities, and increases the difficulty of science popularization work [10, 25]. Besides, it is found that improper planning or tactics of higher education development decreases the effectiveness and successfulness of science popularization, thus higher education institutions should make use of science popularization activities wisely and properly [26]. Current studies mainly focus on the influence mechanism of higher education on science popularization, but it is still not clear how higher education has interactive impacts on science popularization with coordination.

### Effects: Science popularization on higher education

Science popularization also has effects on higher education both positively and negatively. Positively speaking, science popularization itself is a form or approach of education, so the new changes of science popularization contribute to the changes of higher education; in other words, science popularization development facilitates the growth of higher education with more pertinence [6, 27, 28]. Secondly, science popularization activities enhance university students’ passion or motivation for and participation in study, which lead to better performances and competitiveness in higher education [11, 29]. Cases have proved that science popularization is helpful to accelerate the learning outcomes of students in colleges, such as writing skills, literature understanding, physics knowledge acquisition, etc. [11, 30, 31]. Thirdly, science popularization activities help higher education institutions to attract more talented and suitable students; by providing related courses or programs, science popularization activities cultivate students’ understanding of subjects and professions, and those with clear study and career goals are more likely to choose more proper universities so that higher education institutions can indirectly benefit from science popularization activities [32, 33]. Fourth, science popularization benefits tutors in universities or colleges by enhancing their work satisfactions and inspirations; this is mainly because science popularization programs can usually provide research funds and achievability to university tutors [21].

Negatively speaking, science popularization hinders higher education development or competitiveness to some degree. Firstly, science popularization increases gender inequality in higher education systems; it is found that science popularization has different impacts on different genders, and female students are less interested in science popularization activities than male students so that gender imbalance caused by science popularization may occur and higher education competitiveness, which requires gender equality, is likely to decline [10, 34, 35]. Secondly, lacking sufficient focus on science popularization is likely to decrease higher education competitiveness; science popularization activities are good opportunities for students to learn new skills or knowledge, and lacking enough related activities reduces studying chances and competitiveness of college students, which in turn reduces higher education competitiveness [36]. It is also found that some science popularization activities may make students feel boring or lost [29]. Thirdly, some debates or problems may occur in science popularization programs (such as ethical issues, legal issues, social norm issues, etc.), which in turn impede the growth of higher education [37, 38]. Fourthly, venues of science popularization activities, if they are crowded indoor spaces and are less comfortable, are likely to cause working pressures and mental stress of science popularization activity providers from higher education institutions (such as lecturers from universities, volunteers from colleges, etc.) [39, 40].

### Interaction: Higher education and science popularization

In order to assess the coordinated growth between higher education and science popularizaton, we need to detaily explore the interactions between these two systems. It can be found that there are interactions between the two, which is somehow intricate: higher education and science popularization support each other’s development and meanwhile hinder further growth each other. Coupling coordination, namely coordinated growth in this study, is defined to describe the interaction status of two systems which have bilateral impacts each other, and to depict how the systems evolve to more harmonious and synergetic status [41, 42]. Exploring the coordinated growth between higher education and science popularization is important for us to understand their bilateral interactions, however, current studies mainly focus on correlations between higher education and science popularization, the influence mechanism of higher education on science popularization, and the influence mechanism of science popularization on higher education; there are insufficient studies exploring the interaction mechanisms between higher education and science popularization, which is vital to evaluate their coordinated growth status and to take corresponding countermeasures to enhance both systems synchronously; furthermore, studies regarding the comparisons and predications of the coordinated growth between higher education and science popularization temporally and spatially are still relatively insufficient.

To understand the coordinated growth mechanism and growth tendencies between higher education and science popularization is a significant work: it contributes to more effective and specific countermeasures to enhance interactive coordinated growth between the two systems. However, the facts that there are not proper coordinated growth mechanisms and that there are not sufficient or representative indices determine the difficulty of assessing the coordinated growth interactions between the two systems. Besides, former studies ignores inter-regional contrasts and comparisons from both temporal and spatial perspectives, and the indices to assess higher education and science popularization are not universally accepted. Therefore, it is needed to discover the coordinated growth mechanism between the two systems, to determine the indices for more efficient and widely accepted assessment, and to process inter-regional comparisons so that the coordinated growth between higher education and science popularization can be measured and explored.

Therefore, we hereby propose the following research hypotheses:

H1: The overall performances of higher education and science popularization vary temporally and spatially.
H2: Higher education and science popularization can achieve coordinated growth with both temporal and spatial characteristics.

### Research methods selection

Two categories of methods, which are objective category and subjective category, are usually used to assess the weight of indices when measuring the coordinated growth relationship between systems. For the objective category, including cluster analysis method, entropy weight analysis method, main component analysis method, Vlse Kriterijumska Optimizacija Kompromisno Resenje method (VIKOR), ranking of alternatives through functional mapping of criterion sub-intervals into a single interval method (RAFSI), criteria importance through inter-criteria correlation method (CRITIC), etc., the methods obtain weights of indices by calculating data objectively, therefore, the weights are much more objective with less bias or errors; however, some problems will be encountered if we use these objective methods; for instance, some important indices will be eliminated if main component analysis method is solely used; the accuracy is doubtful if cluster analysis method is used solely without combing other methods; VIKOR requires the accuracy of weight coefficients of the criterion, which in practice is somehow difficult to obtain; entropy, CRITIC, and RAFSI may not correct and may bias the weights in certain multi-criteria decision-making cases if they are used solely [43–47].

For the subjective category, including analytic hierarchy process method, expert scoring method, the best-worst method (BWM), full consistency method (FUCOM), level based weight assessment method (LBWA), etc., the methods obtain weights of indices based on personal preferences, therefore, the weights are more likely to reflect the real or preferred ideal situations; besides, the subjective methods can be applied in various fields and thus are welcomed in certain studies; however, the limitations of the subjective methods restrict the further use and make the results less trustful; for instance, BWM is difficult to measure the differences between grades among indices; FUCOM and LBWA are subject to score markers whose personal bias may distort the weight of indices [48–51].

The combination of entropy weight analysis method and TOPSIS method, namely Technique for Order Preference by Similarity to Ideal Solutions method, is much better in weighting the indices and evaluating the coordinated growth between systems. The entropy weight analysis method can be applied to objectively assess the weights of indices by measuring the stability of indices and the system, and the TOPSIS method can be applied to assess the relative importance of the alternatives or options [41]. The joint use of these two methods has been widely accepted and utilized in measuring coordinated growth relations between systems (such as between economy and environment, between urbanization and economy, etc.), and has been proved successful in evaluating the coordination status in various fields [52, 53]. The combination of the two methods guarantees objectivity of results meanwhile avoids potential problems of using solely method, and is novelty in analyzing the coordinated growth relations between the two study objects (higher education and science popularization).

### Research case

**Fig 1:**
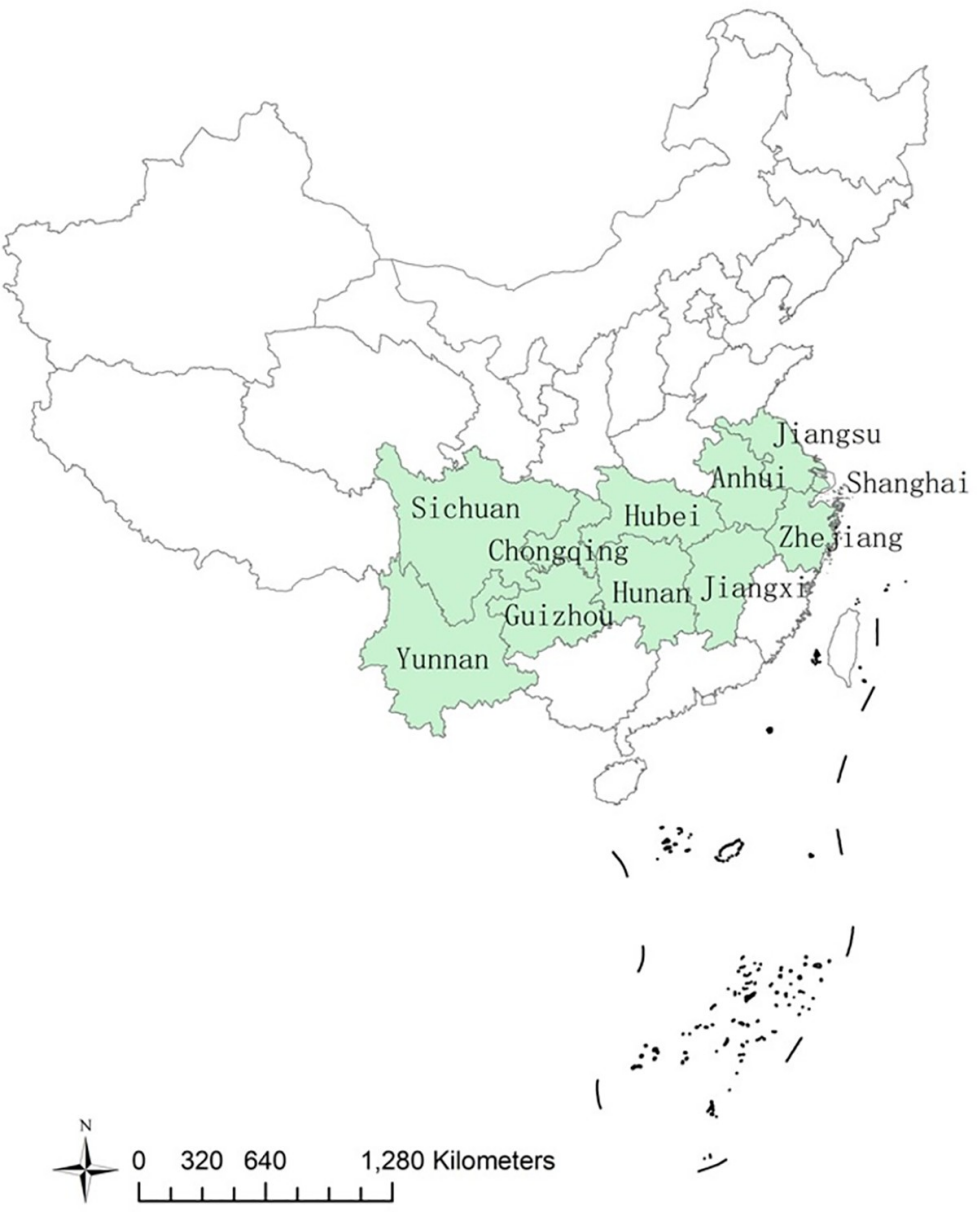
Research case.

In this study, we choose the 11 provincial regions of the Yangtze River Economic Belt in China as research case (Fig 1). The Yangtze River Economic Belt is a representative case mainly because of the national strategy to develop the Yangtze River Economic Belt with integrated actions and because of its great variations of both higher education and science popularization among regions. For instance, for the higher education system, the higher educational fund of Jiangsu is about twice that of Guizhou, and the number of higher education institutions of Shanghai is about 4 times that of Chongqing; for the science popularization system, the exhibition areas of science and technology halls of Zhejiang are 4 times that of Yunnan, and the annual science popularization fund of Shanghai is 4.6 times that of Guizhou. The differences of higher education and science popularization among regions are obvious and have caused inter-regional imbalance problems, therefore, it is necessary to study how to achieve coordinated growth between the two systems among regions. Selecting provincial regions of the Yangtze River Economic Belt as cases is proper to explore the temporal-spatial variations of higher education and science popularization, to discover the coordinated growth mechanism between higher education and science popularization, and to provide examples to other countries sharing similarities to achieve coordinated growth between the two systems.

## Methods

### Research process

**Fig 2:**
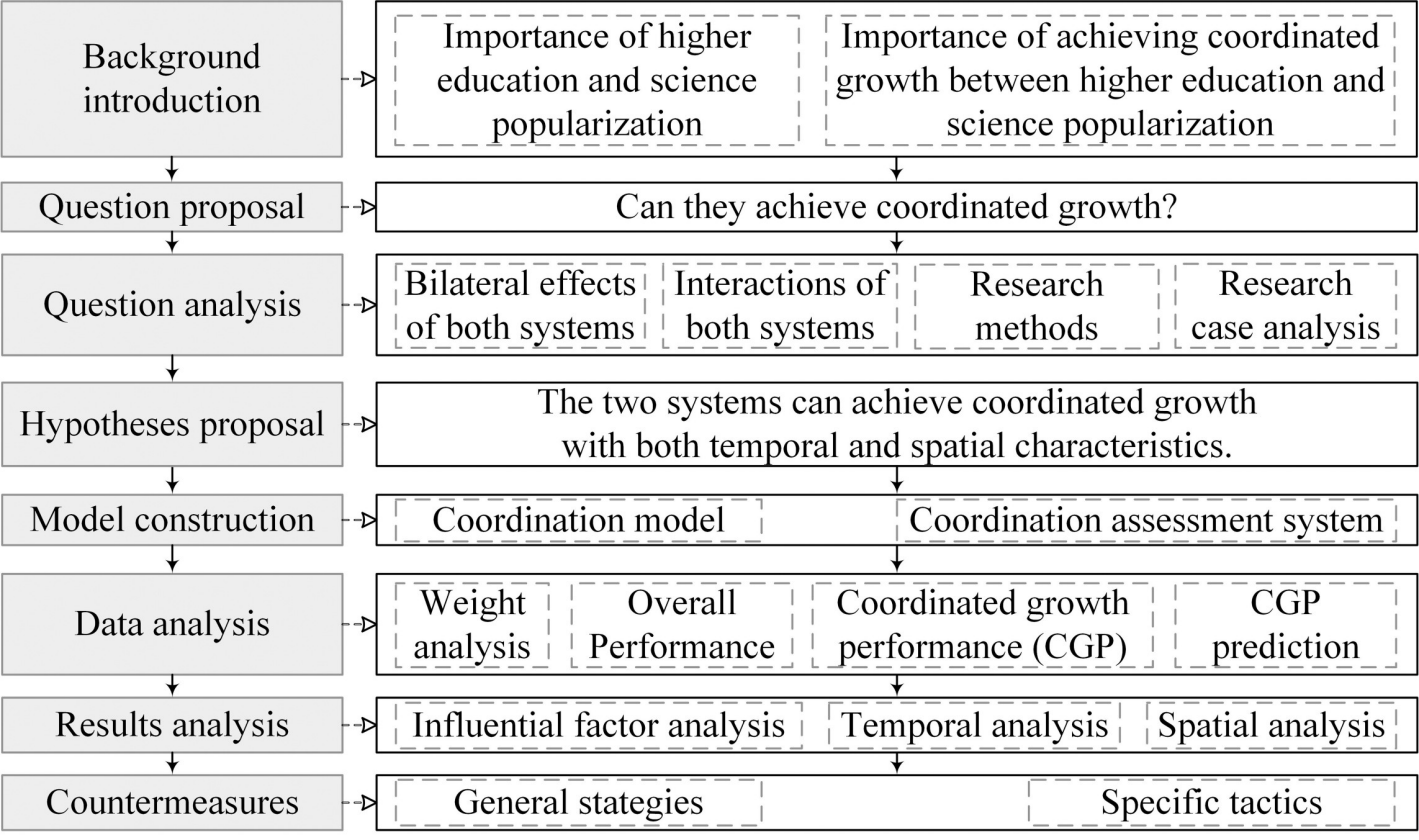
Flowchart of the research process.

The flowchart of the research processes is shown in Fig 2. In detail, this paper starts by introducing the background of this research, and by clarifying the purpose of this research. Then, by doing literature review, this paper analyses the research question theoretically and qualitatively, proposes hypotheses, and constructs the coordination model and the coordination assessment system based on the theoretical analysis results. Furthermore, using entropy weight analysis method, Technique for Order Preference by Similarity to Ideal Solutions method (TOPSIS method), and GM(1,1) gray prediction method, this paper analyzes the main influential factors, and both temporally and spatially discusses the overall performances of both higher education and science popularization, and coordinated growth performances together with the tendencies. Finally, the countermeasures to enhance the coordinated growth between the two systems are proposed based on the discussion results.

### Coordination model

There are intricate interactions between higher education and science popularization, and both factors make up an organic system where the two interact and coordinate each other. On the one hand, devotion of personnel and money into higher education industry contributes to the growth of higher education institutions, which in turn provides much support to future science popularization activities (such as providing labors and experts to support science popularization activities); besides, the output of higher education activities, such as scientific papers, scientific publications, etc., provides abundant information and materials to science popularization so that its performance can be enhanced. However, as the total resources of society are limited, more input to higher education means less input to science popularization, therefore, the scales and performances of science popularization are hindered if more resources or inputs are given to higher education; also, there are certain controversial higher education related outputs (such as scientific patents or projects) which are likely to have negative impacts on science popularization performances if being used improperly or unethically.

On the other hand, larger scale of science popularization (such as larger size of exhibition halls of science popularization and more numbers of science and technology museums) usually means more spaces for higher education activities and practices, which plays as an input role in supporting higher education growth; what is more, by cultivating potential scientists or specialists who will participate in future higher education development, science popularization facilitates higher education input and output. However, by devoting more budget and hiring more talented labors, more resources are occupied by science popularization industry, which to some degree retards the input of higher education; besides, poor performances of science popularization also decrease the attractiveness of science and technology from college students, reduce the competitiveness of higher education, and impede its potential of high qualified growth.

**Fig 3:**
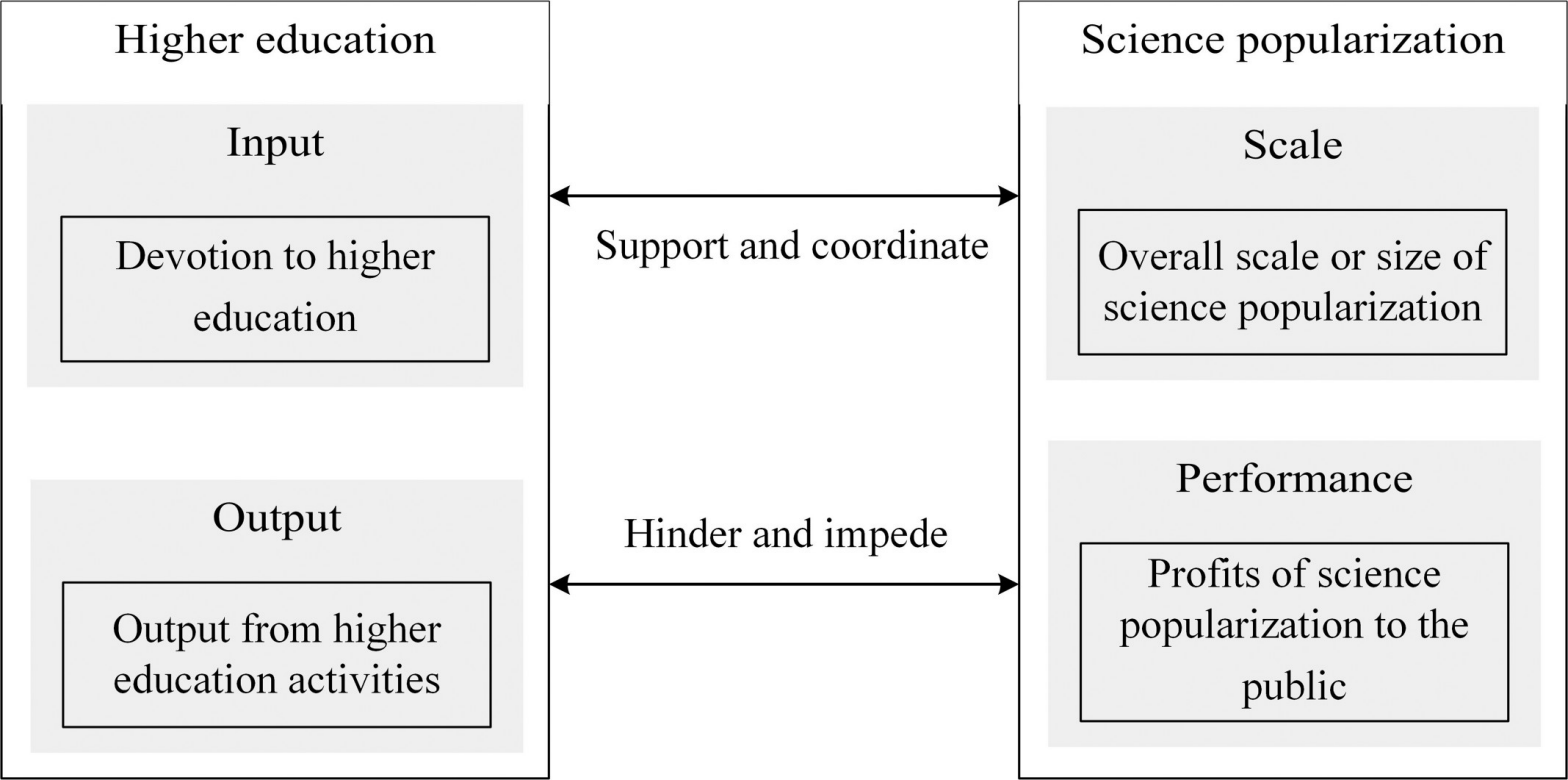
Coordination model.

Therefore, higher education and science popularization are interacting each other with coordinated growth relations; based on that, we construct the coordination model, which is shown in Fig 3, and which is valid and effective to explore the coordinated growth interaction mechanism of higher education and science popularization, and to assess the coordinated growth status between these two systems.

### Coordination assessment system

Based on the coordination model, we construct the coordination assessment system and select indices, which are mainly screened from previous literature, to empirically measure the overall performance status of the two systems respectively, and the coordinated growth status between the two systems.

Several rules are applied during the index’s selection process: (1) the literature should be high qualified with peer review process (indexed in SCI or SSCI is preferred); (2) the literature should be quantitative, meaning the indices can be calculated; (3) the indices have been adopted and cited as wide as possible in the academia; (4) the data of the indices are open access and easy to obtain; (5) the indices are clear and easy to understand for everyone; (6) the indices are aggregated to reflect the key aspects or components of higher education and science popularization; (7) the indices have solved the problem of multi-collinearity [54, 55]. We proceed the qualitative analysis, coefficient analysis and significance tests, then select 8 indices for higher education system (with 2 aspects) and 6 indices for science popularization system (with 2 aspects), and finally set up the higher education-science popularization coordination assessment system to assess the coordinated growth relations between the two systems.

The higher education system is composed of two aspects, namely higher education input and higher education output. The aspect of higher education input, which reflects the resource devotion into higher education industry, is composed of 5 indices; in detail, the indices of the number of teachers, the number of students, and the annual budget, and the number of higher education institutes reflect the input quantity from the human resources, financial, and existence carrier perspectives [3, 56]; the index of the annual budget per student reflects the input quality by evaluating the ratio of financial perspectives [7, 56]. The aspect of higher education output, which reflects the outcomes of higher education activities, is composed of 3 indices; in detail, the number of publications, the number of science and technology projects, and the number of scientific papers reflect the overall output of scientific activities from higher education institutes; these indices have been selected in previous highly cited and recognized studies and have been widely accepted to efficiently and effectively to assess higher education output [3, 7].

The science popularization system is also composed of two aspects, namely science popularization scale and science popularization performance. The aspect of science popularization scale demonstrates the overall scale of science popularization industry and related participants, including 4 indices: the number of personnel, the number of visitors, the number of science and technology museums, and the number of exhibition area of museums; these 4 indices are representative in demonstrating the scale of science popularization industry together with its activities [57]. The aspect of science popularization performance demonstrates the overall performances of science popularization. There are 2 indices, which are the number of science popularization activities and the annual budget of science popularization industry, to assess the number of science and technology week held and the funding for science and technology popularization; these indices, considering data availability, are also selected from the highly cited literature after careful screening, and are representative in illustrating the performances of science popularization activities [57].

The details of the coordination assessment system are shown in Tables 1 and 2.

**Table 1:** Coordination assessment system for higher education.

Aspects	Indices	Explanations
Input	Number of teachers	To measure the input quantity of teachers in higher education institutes
	Number of students	To measure the input quantity of enrolled students in higher education institutes
	Annual budget	To measure the financial input of higher education industry
	Number of higher education institutes	To measure the input quantity of higher education institutes
	Annual budget per student	To measure the input quality from the fiscal perspective
Output	Number of publications	To measure the overall output of scientific publications
	Number of science and technology projects	To measure the overall output of science and technology projects
	Number of scientific papers	To measure the overall output of scientific papers

**Table 2:** Coordination assessment system for science popularization.

Aspects	Indices	Explanations
Scale	Number of personnel	To measure the scale of full-time science popularization personnel
	Number of visitors	To measure the scale of participants to science popularization venues
	Number of science and technology museums	To measure the overall number of science popularization venues
	Number of exhibition area of museums	To measure the scale of science popularization activity areas
Performance	Number of activities	To measure the performance of science popularization activities
	Annual budget	To measure the fiscal performance of science popularization industry

### Analysis process

By jointly using entropy weight analysis method and TOPSIS method, we firstly obtain the overall performances of the two systems respectively, secondly obtain the coordinated growth performances between them, and thirdly predicted the coordinated growth performances in the near future. The 9 years’ data of this study (2010–2018) are selected from China Statistical Yearbook on Science and Technology, China Statistical Yearbook, and Compilation of Statistics on Science and Technology of Higher Education Institutions due to data accessibility. These two files are published by the national authority of China; therefore, the objectivity and authenticity of the data is guaranteed.

(1) Standardize data for later calculation procedures. For the matrix *x*_*ij*_, j is the index and i is the option. We select 30 regions with 9 years as the study case, so there are 270 options in total in this study. We use formula (1) to obtain the standardized data $x_{ij}^{\prime }$, where *i* = 1,2,…,n; *j* = 1,2,…,m. Then we can obtain the maximized value and minimized value $\underset{1\leq j\leq m}{max}x_{ij}$ and $\underset{1\leq j\leq m}{min}x_{ij}$ of the standardized data matrix respectively.


$$x_{ij}^{\prime }=\frac{x_{ij}}{\sum_{i=1}^{m}x_{ij}}$$ (1)

(2) Use formula (2) to obtain weight w of the index j with entropy weight analysis method. *ln f*_*ij*_ is to assure the significance and $f_{ij}=\frac{1+x_{ij}^{\prime }}{\sum_{i=1}^{n}\left(1+X_{ij}^{\prime }\right)}$.


$$w_{j}=\frac{1-[-(\sum_{i=1}^{n}f_{ij}\;\ln\;fij)]}{m-\sum_{j=1}^{m}\left[-(\sum_{i=1}^{n}f_{ij}\;\ln\;fij)\right]}$$ (2)

(3) Use formula (3) to obtain overall performance (OP) with TOPSIS method. Here $A=(\underset{1\leq i\leq n}{max}x_{i1},\underset{1\leq i\leq n}{max}x_{i2},\ldots ,\underset{1\leq i\leq n}{max}x_{im})$ and $B=(\underset{1\leq i\leq n}{min}x_{i1},\underset{1\leq i\leq n}{min}x_{i2},\ldots ,\underset{1\leq i\leq n}{min}x_{im})$.


$${OP}_{i}=\frac{\sqrt{\sum_{j=1}^{m}w_{j}{\left(x_{ij}-B_{j}\right)}^{2}}}{\sqrt{\sum_{j=1}^{m}w_{j}{\left(x_{ij}-A_{j}\right)}^{2}}+\sqrt{\sum_{j=1}^{m}w_{j}{\left(x_{ij}-B_{j}\right)}^{2}}}$$ (3)

(4) Set up the assessment grade of the overall performance. The value of the overall performance ranges from 0 to 1, and we classify the grades of the overall performance based on the equal interval principle, with the details exhibited in Table 3 [42]. In specific, there are five grades, namely excellent (above 0.8), fair (0.6 to 0.8), average (0.4 to 0.6), acceptable (0.2 to 0.4), and unacceptable (below 0.2).

**Table 3:** Assessment grade of the overall performance.

Assessment Grade	Interval
Unacceptable	OP < 0.2
Acceptable	0.2 ≤ OP < 0.4
Average	0.4 ≤ OP < 0.6
Fair	0.6 ≤ OP < 0.8
Excellent	OP ≥ 0.8

(5) Obtain coordinated growth performance (CGP). Here the coupling coordination degree model is used. OP(h) is the overall performance of the higher education system and OP(s) is the overall performance of the science popularization system. Besides, the two systems (higher education and science popularization) are equally important in interacting within the coordinated growth mechanism, so the coefficients of the two systems φ and γ are both equal to 0.5 [54].


$$CGP=\sqrt{{\left\{\frac{OP(h)\times OP(s)}{{\left(\frac{OP(h)+OP(s)}{2}\right)}^{2}}\right\}}^{\frac{1}{2}}\times [\varphi OP(h)+\gamma OP(s)]}$$ (4)

(6) Set up the assessment grade of the coordinated growth performance. The value of the coordinated growth performance also ranges from 0 to 1, and we classify the grades based on the equal interval principle, too. The details are shown in Table 4.

**Table 4:** Assessment grade of the coordinated growth performance.

Category	Range	Assessment Grade
Non-coordination	0.0 ≤ CGP < 0.1	Extreme incoordination
	0.1 ≤ CGP < 0.2	Unfair incoordination
	0.2 ≤ CGP < 0.3	Average incoordination
	0.3 ≤ CGP < 0.4	Unacceptable incoordination
Transitioning	0.4 ≤ CGP < 0.5	Transitional incoordination
	0.5 ≤ CGP < 0.6	Transitional coordination
Coordination	0.6 ≤ CGP < 0.7	Acceptable coordination
	0.7 ≤ CGP < 0.8	Average coordination
	0.8 ≤ CGP < 0.9	Fair coordination
	0.9 ≤ CGP ≤ 1.0	Excellent coordination

(7) In this study we use GM (1,1) gray prediction method to analyze the tendency of the coordinated growth performance of the two systems. GM (1,1) is a more preferred method because it can be effective in predictions with relatively finite samples [58], which is what this study encounters. In detail, for series *X*_0_ = {*x*_0_(1),*x*_0_(2),⋯*x*_0_(*m*)} (m: observational option), we obtain the new series *X*_1_ = {*x*_1_(1),*x*_1_(2),⋯*x*_1_(*m*)} via $x_{1}(t)=\sum_{i=1}^{t}x_{0}(i)$; we obtain the differential equation with $\lambda =\frac{dx_{1}(t)}{dt}+\alpha x_{1}(t)$ (*λ*: endogenous control gray value; α: development gray value; *t* = 1,2,⋯,*m*). Then we obtain $\hat{a}=(\frac{\alpha }{\lambda })={\left(B^{T}B\right)}^{-1}B^{T}Y\;(\hat{a}:$ estimated parameter vector; *B* = [−*Z*_1_(2),−*Z*_1_(3),⋯,−*Z*_1_(*m*), 1,1,⋯1]^*T*^; *Y* = [*x*_0_(2),⋯,*x*_0_(*m*)]^*T*^), assess the differential equation, and obtain the prediction model.


$$\hat{x}(t+1)=\left[x_{0}(1)-\frac{\lambda }{\alpha }\right]e^{-\alpha t}+\frac{\lambda }{\alpha }$$ (5)

**Fig 4:**
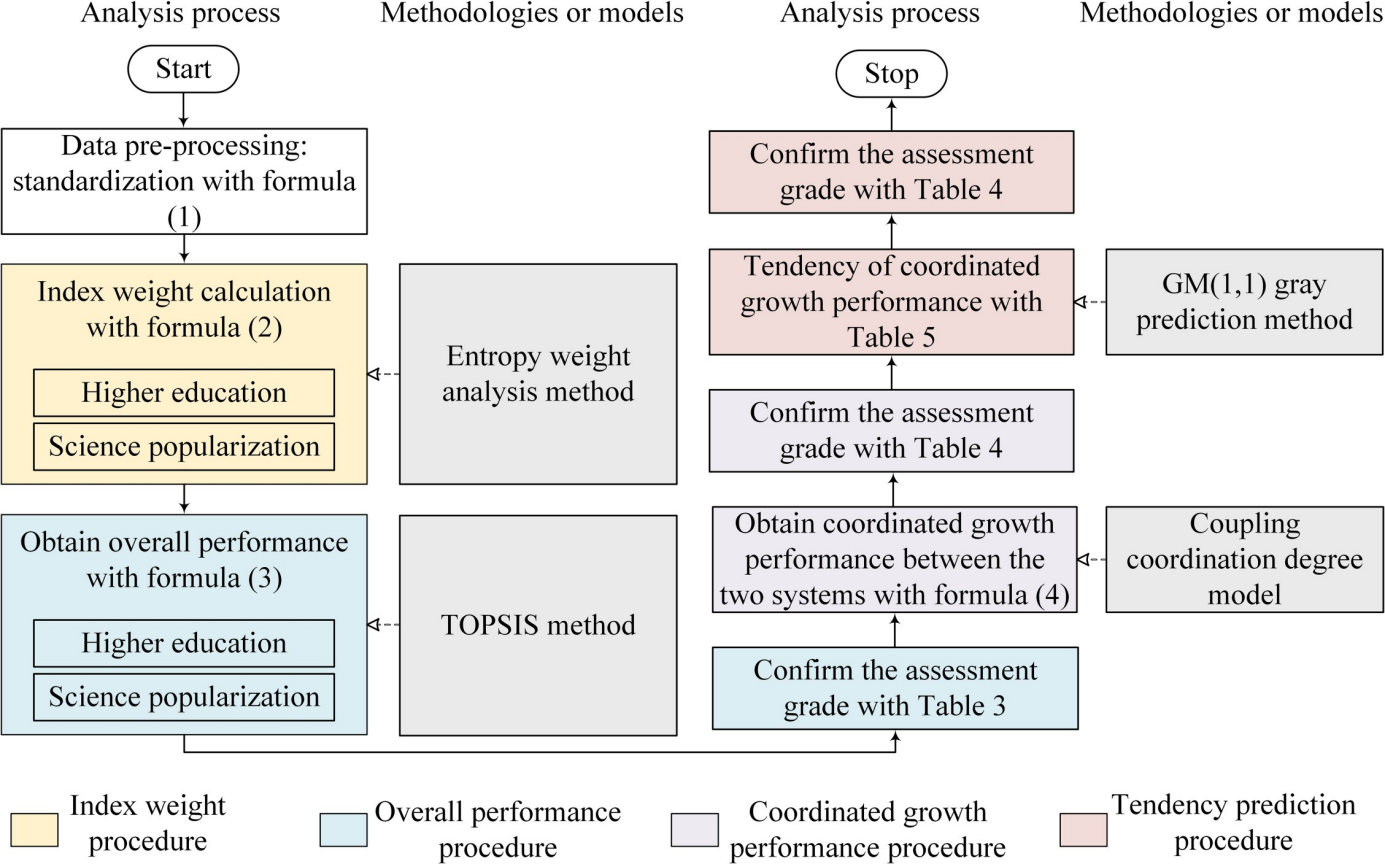
Flowchart of the analysis processes.

If *a*≤0.3, we can use the data to predict the tendency; *a* is the most common parameter to assess the prediction model’s precision [41]. Besides, we obtain the residual difference $\varepsilon_{0}(t)=x_{0}(t)-{\hat{x}}_{0}(t)$ and relative error $q(t)=\frac{\varepsilon_{0}(t)}{x_{0}(t)}\times 100\%$ to reassure the precision of the model. Then we obtain ${\bar{\varepsilon }}_{0}=\frac{1}{m-1}\sum_{t=2}^{m}\varepsilon_{0}(t),\;S_{\varepsilon }^{2}=\frac{1}{m-1}{\sum_{t=2}^{m}\left(\varepsilon_{0}(t)-{\bar{\varepsilon }}_{0}\right)}^{2},\;{\bar{x}}_{0}=\frac{1}{m-1}\sum_{t=2}^{m}x_{0}(t)$, and $S_{x}^{2}=\frac{1}{m-1}{\sum_{t=2}^{m}\left(x_{0}(t)-{\bar{x}}_{0}\right)}^{2}\;({\bar{\varepsilon }}_{0}$ and ${\bar{x}}_{0}$: mean of ε_0_(*t*) and x_0_(*t*) respectively; $S_{\varepsilon }^{2}$ and $S_{x}^{2}$: variance of ε_0_(*t*) and x_0_(*t*) respectively), and assess $P=p(|\varepsilon_{0}(t)-{\bar{\varepsilon }}_{0}|<0.6745S_{x})$ to reassure the precision of the model again (*P*: small error probability). The data are accurate for prediction if the residual difference *r* is no less than 0.6, the relative error *a* is no large than 0.2, and the small error probability *P* is no less than 0.6 [41]. The detailed flowchart of the analysis processes is shown in Fig 4 [59].

## Results and discussions

### Index weight analysis

The weights of the indexes are shown in Table 5. It is clear to see that in the higher education system, the index of the annual budget per student has the highest weight (0.1994), followed by the number of publications and the number of scientific papers (0.1810 and 0.1661), demonstrating that these three are the main influential factors of higher education system. Besides, in the science popularization system, the index of the number of science and technology museums has the highest weight (0.2935), followed by the indexes of the number of visitors (0.2530) and the number of exhibition area of museums (0.1611), demonstrating their importance in influencing the development of science popularization. The results are partly supported by former studies, which also supports the idea that the education budget and the quantity of science and technology museums affect each system significantly [5, 60].

**Table 5:** Index weight.

System	Aspects	Indices	Weights
Higher education	Input	Number of teachers	0.0726
		Number of students	0.0690
		Annual budget	0.0775
		Number of higher education institutes	0.0939
		Annual budget per student	0.1994
	Output	Number of publications	0.1810
		Number of science and technology projects	0.1404
		Number of scientific papers	0.1661
Science popularization	Scale	Number of personnel	0.0632
		Number of visitors	0.2530
		Number of science and technology museums	0.2935
		Number of exhibition area of museums	0.1611
	Performance	Number of activities	0.0752
		Annual budget	0.1540

### Overall performance analysis

**Fig 5:**
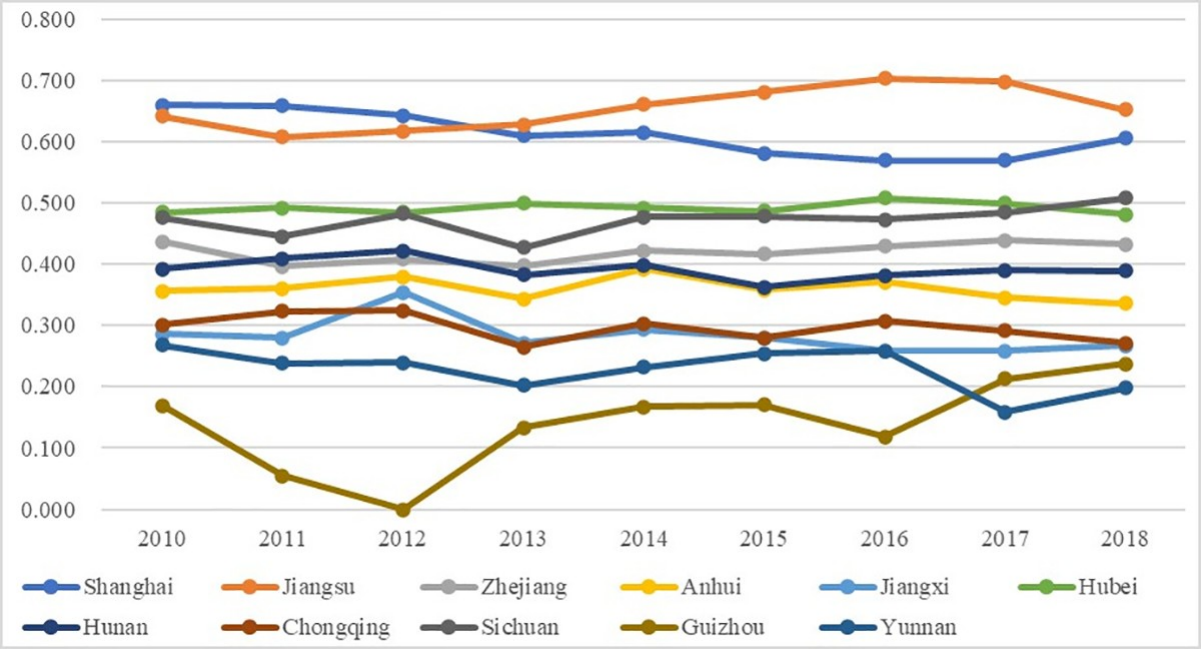
Overall performance of the higher education system.

Fig 5 exhibits the temporal dynamic changes of the overall performance of the higher education system, with the detailed numbers shown in S1 Table. Generally, the 9 yeas’ overall performances of higher education for most regions remained relatively stable with mild fluctuations, and the regions could be divided into 4 assessment grades according to Table 3. The first grade is the Unacceptable Assessment Grade (OP<0.2), including Guizhou, which witnessed an obvious decline from 2010 to 2012, demonstrating that the overall performances of higher education of Guizhou were less satisfying. The second grade is the Acceptable Assessment Grade (0.2–0.4), including most regions (5 regions); among them, Yunnan had an obvious decline to a lower grade for the recent continuous years, proving the deteriorative performances in higher education. The third grade is the Average Assessment Grade (0.4–0.6), including 3 regions (Zhejiang, Hubei, and Sichuan), and they remained stable fluctuations within this grade for most years, demonstrating the average and unglamorous performances of these regions. The fourth grade is the Fair Assessment Grade (0.6–0.8), including 2 regions (Shanghai and Jiangsu), proving the relatively better performances of these places in the higher education system. The results are mainly consistent with former studies that higher education performances were mildly fluctuating [3], while there is a new finding that the overall performances can reach 0 (e.g., Guizhou in 2012), mainly because this study selects more abbreviated and represented indices to depict the actual overall performances more precisely, and uses entropy weight analysis and TOPSIS method which evaluate the relative “better” or “worse” performances [55].

**Fig 6:**
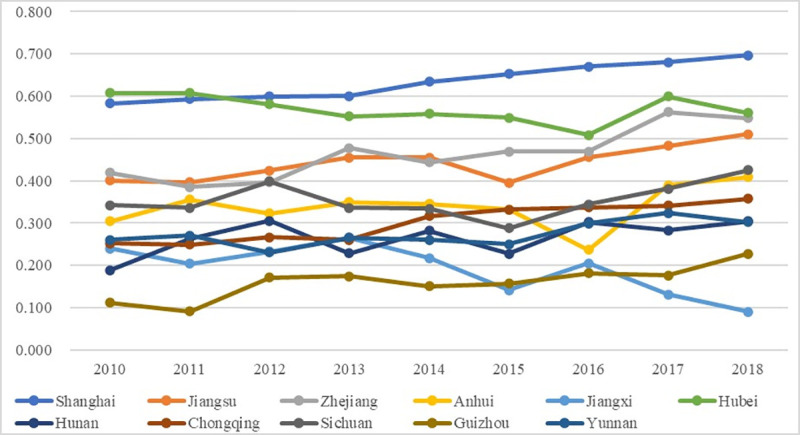
Overall performance of the science popularization system.

Fig 6 exhibits the dynamic changes of the overall performances of the science popularization system for 9 years, with the detailed numbers exhibited in S2 Table. Generally, there are several interesting findings: firstly, the increasing tendencies of the science popularization system were more obvious than the higher education system; secondly, the differences of the overall performances among regions were gradually expanding these years. In specific, the regions could also be divided into four grades. The first one is the Unacceptable Assessment Grade (0–0.2), including 2 regions (Jiangxi and Guizhou); Guizhou fell into the Unacceptable Assessment Grade for both systems, proving that it performed poor in both systems. The second one is the Acceptable Assessment Grade (0.2–0.4) with the majority of regions included (5 regions in total), which proved the overall average performances of science popularization in the Yangtze River Economic Belt. The third one is the Average Assessment Grade (0.4–0.6), including 3 regions (Jiangsu, Zhejiang, and Hubei); among them, 2 regions had significant increase such as Jiangsu and Zhejiang, proving their enhancing performances in science popularization. The fourth one is the Fair Assessment Grade (0.6–0.8), including 1 region only (Shanghai). Shanghai witnessed an apparent increase from the Average Assessment Grade (0.4–0.6) to the Fair Assessment Grade (0.6–0.8), illustrating its growth potential in this system. The overall performances of the science popularization system are likely to increase more drastically mainly because of the large or substantial annual increases of certain factors, such as the number of science popularization activities, the number of visitors to science popularization venues, and the budget for science popularization activities. This is supported by previous work that devotions of science popularization resources from various aspects contribute to science popularization growth [61].

**Fig 7:**
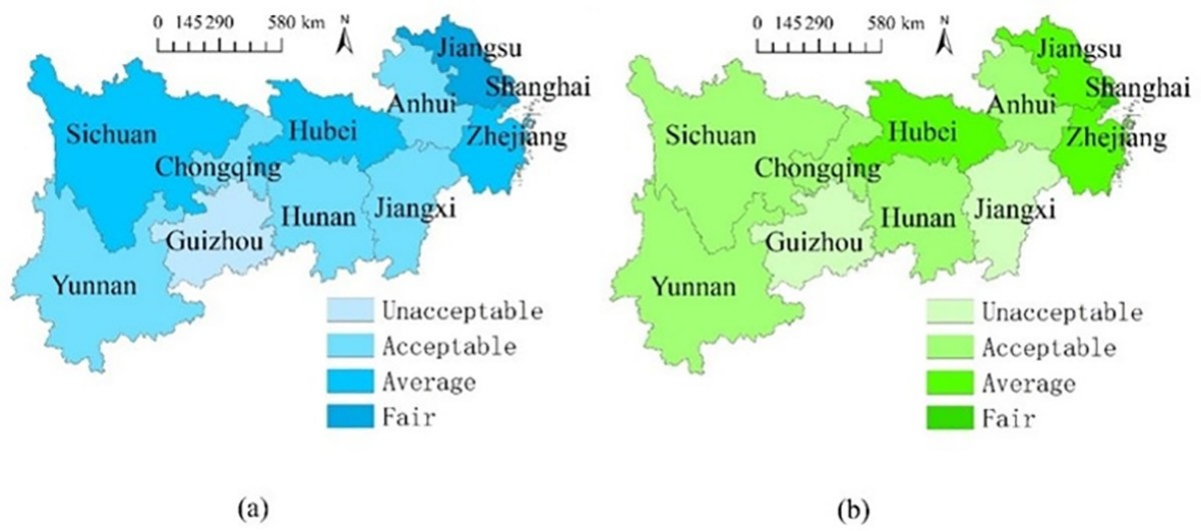
Spatial comparisons of the average overall performances. (a) Higher education system; (b) Science popularization system.

Fig 7 compares the spatial differences of the average overall performances between higher education system and science popularization system. There are some general findings: there are similarities of the spatial distributions of the two systems, and the average overall performances of higher education are relatively higher than those of science popularization system, demonstrating that higher education performances are spatially related to science popularization performances, and higher education in the Yangtze River Economic Belt performs relatively better than science popularization. This is a new finding with no previous studies revealing this; such difference can be interpreted by the tradition of Chinese culture where education is always highly emphasized, and more resources are likely to be allocated in education [62]; besides, it is more possible for higher education system to use science popularization in order to enhance its overall performance, while there are comparatively fewer opportunities for science popularization system to use higher education to enhance the performances, which is also supported by former studies [63, 64]. In specific, the spatial distributions of the average overall performances of the two systems show slight differences: (1) for the higher education system, the generally believed regions with advantages in education (Sichuan, Hubei, Jiangsu, Shanghai, and Zhejiang) still maintained the competitiveness in higher education. These places enjoyed abundant population and traditionally paid great attention to education, therefore, they enjoy qualified local higher education institutes and college students with quantity, and performs better than other regions, which is supported by previous work [7]; (2) for the science popularization system, the coastal regions had better average overall performances (Jiangsu, Shanghai, and Zhejiang), mainly because of the convenient transportation and strong fiscal strength so that they were relatively much easier to attract more visitors, construct more science and technology museums, and organize more relevant activities, which is also supported by previous work [43].

### Coordinated growth performance analysis

**Fig 8:**
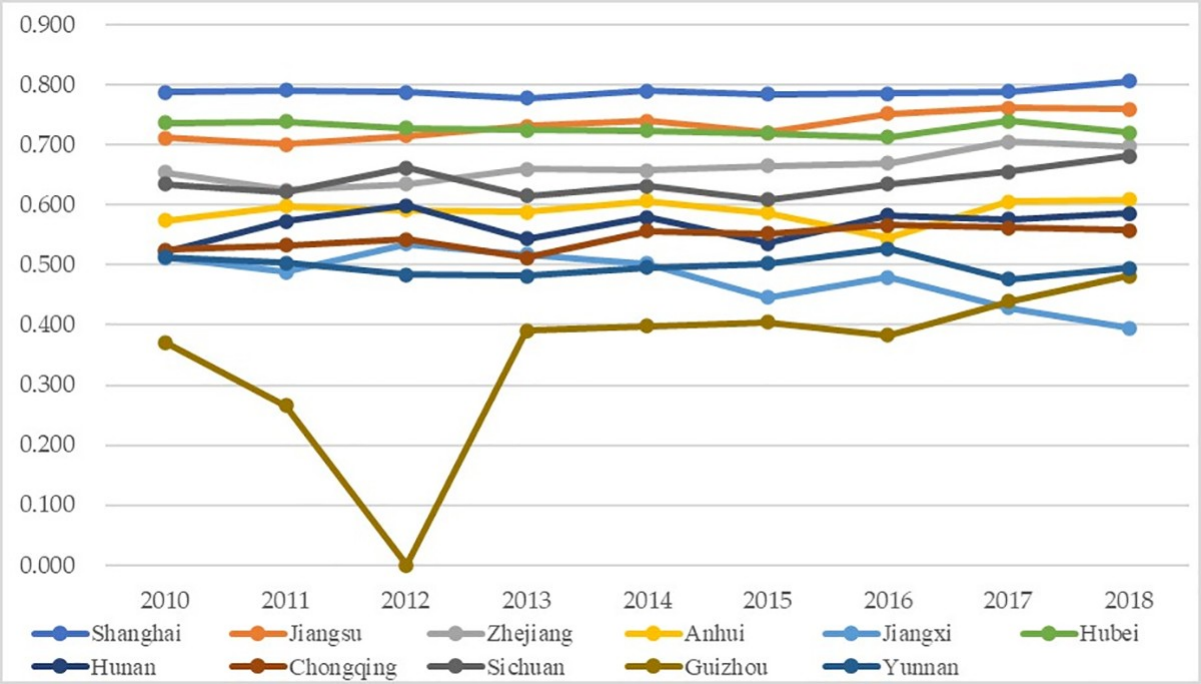
Coordinated growth performance between higher education and science popularization.

Fig 8 exhibits the temporal changes of the coordinated growth performance between higher education and science popularization, with the detailed numbers exhibited in S3 Table. Generally, the coordinated growth performances of most regions remained mild fluctuations, demonstrating that the higher education system and the science popularization system kept relatively stable coordinated status. In specific, according to the assessment grade (Table 4), most regions were above the Transitional Coordination Assessment Grade (above 0.5), demonstrating that the two systems kept relatively stable coordinated status in these places; among them, half were in the transitioning category (0.4–0.6), demonstrating that these places were in the transitional periods and more efforts were encouraged to improve the coordinated growth performances into the coordination range. Besides, Shanghai was note-worthily outstanding in the coordinated growth performance as its values were always higher than other places among all years (about 0.8), whereas Guizhou is the apparent opposite: it was obviously lower in the coordinated growth performance than other places (below 0.5), thus corresponding countermeasures should be required to enhance its values of coordinated growth performance and to avoid the recurrence of the extreme incoordination status in 2012, which was mainly because all the indices of higher education in Guizhou in 2012 indicated its worst performance among the 11 regions and thus led to unacceptable performance in the higher education system that year. The mild fluctuations and the benign interactions between systems can also be observed in previous work, believing such mild fluctuations demonstrate the benign coordinated growth among the systems [55, 65]; for instance, the interactions among the factors of childhood education also keep such benign coordination interactions [66].

**Fig 9:**
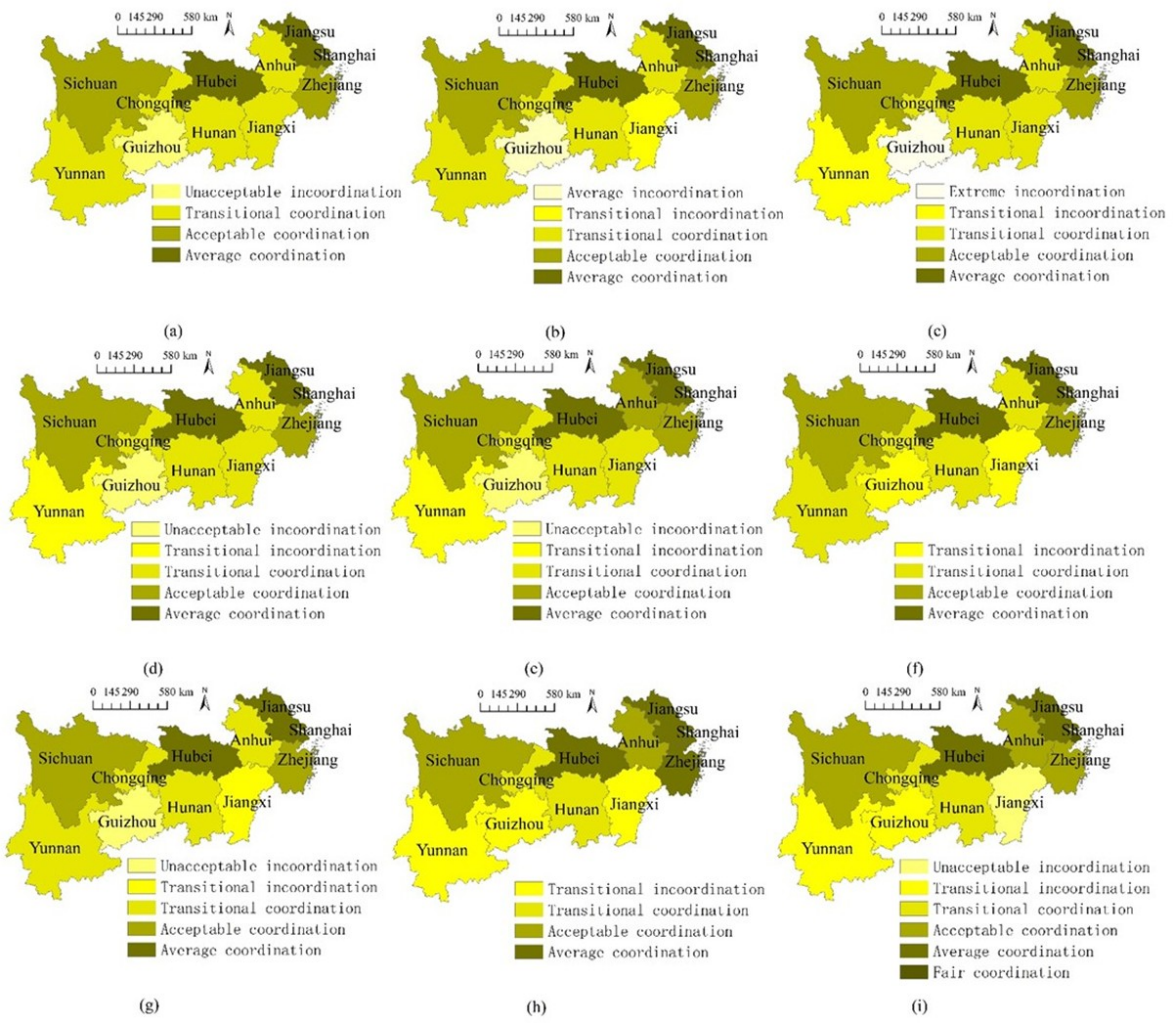
Spatial comparisons of the coordinated growth performance. (a-i) 2010–2018.

The dynamic spatial changes of the coordinated growth performance between the systems are exhibited in Fig 9. There are several new findings regarding the spatial distributions of the coordinated growth performance. (1) The coastal regions had generally better coordinated growth performances than other places. Another particular case is Hubei, which is in the middle area of the Yangtze River Economic Belt. In detail, the coastal regions (Jiangsu and Shanghai) and Hubei had better performances mainly because of the adequate budget for both higher education and science popularization, large amount of output in higher education, large scale of science popularization venues, and high qualified stakeholders (such as teachers, students, personnel, and visitors) of the two systems. Such spatial differences appear in former studies; for instance, spatial differences exist when analyzing the coupling coordination relations between carbon emission and eco-environment [67]. (2) Though there were gaps compared with other places, the western regions (especially Guizhou) were accelerating their coordinated growth performances and were gradually reducing the gap. Guizhou gradually increased from the less coordinated grade (Extreme Incoordination Assessment Grade) to the more coordinated one (Transitional Coordination Assessment Grade), mainly because of the obvious increasing fiscal devotion to higher education and the comprehensive development of the science popularization system. Besides, the declining gaps between Guizhou and the rest places mainly benefited from several national planning and strategies. For instance, after the initiation of certain national plans such as Outline of China’s National Plan for Medium and Long-term Education Reform and Development, the Thematic Planning for Higher Education, the Outline of the Action Plan for the Nation’s Science Literacy, and the 13th Five-Year Plan for national Science Popularization and Innovative Culture Construction, Guizhou had accelerating growth and gradually reduce the differences with other regions. This finding is consistent with former studies where national strategies aiming to enhance national overall high-quality development contribute to more balanced status among regions if the strategies are executed properly [68, 69]. (3) With the passage of time, it is possible for the coordinated growth performance to decrease. For instance, Yunnan and Jiangxi encountered obvious declines of the coordinated growth performances from more coordinated statuses to less coordinated ones, demonstrating there were bilateral blocking mechanism during the coupling coordination interactions between the two systems; this is supported by previous work: some factors in education may hinder the growth of science career [65], thus lag behind the coordinated growth between systems. Therefore, specific countermeasures should be used to stop this decline tendency.

### Coordinated growth performance prediction

The details of the predictions of the coordinated growth performances from 2019 to 2021 are shown in S4 Table. The data of Guizhou in 2012 (0.000) is eliminated in the prediction calculations due to the biased effect of extreme values. GM (1,1) requires at least a less than 0.3, P no less than 0.6, or r larger than 0.6 for alternatives in order to accurate predict tendencies, and all alternatives meet the criteria, pass the accuracy test, and are capable of prediction process.

**Fig 10:**
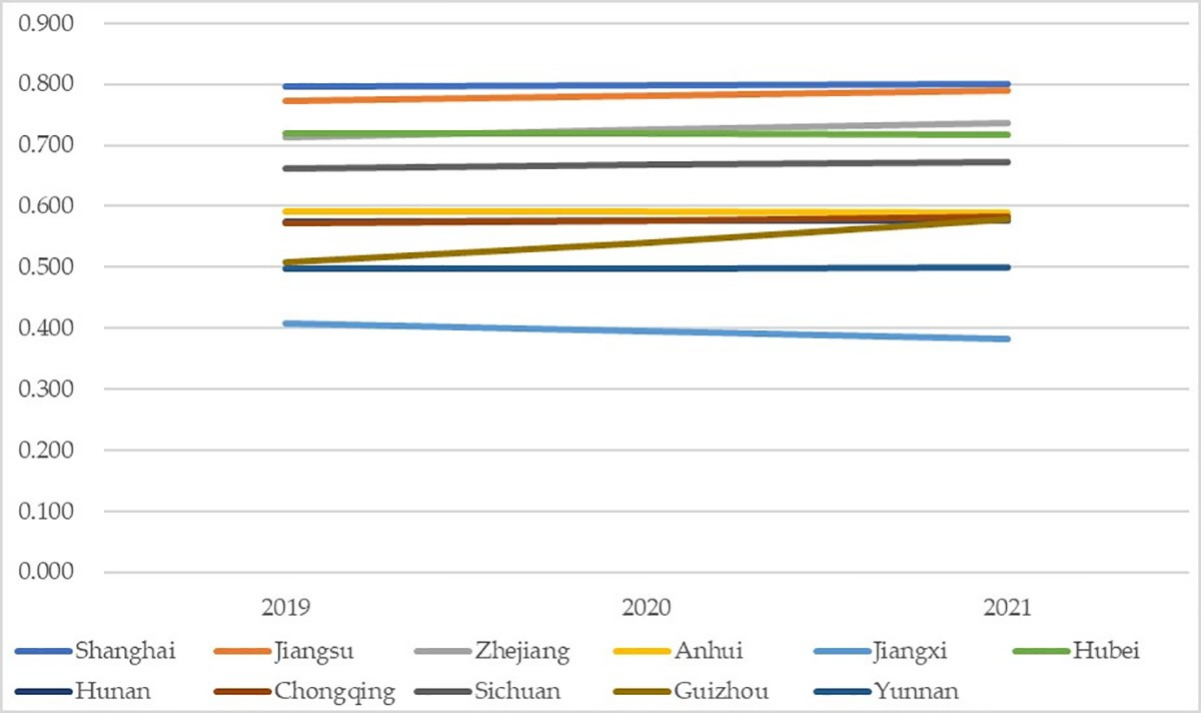
Coordinated growth performance prediction.

The predicted dynamic changes of the coordinated growth performances are shown in Fig 10. Generally, the predicted changes of most regions will remain stable (even more stable than in the past years) with slight increase; the most outstanding one is Guizhou, which will increase its value greatly from 0.507 to 0.578 within the Transitional Coordination Assessment Grade (0.5–0.6). Such increase demonstrates that the higher education system and the science popularization system have taken positive, interactive, and coordinated effects. There is also an exception: Jiangxi will encounter obvious declines from the Transitional Incoordination Assessment Grade (0.4–0.5) to the Unacceptable Incoordination Assessment Grade (0.3–0.4), proving that the two system will hinder and restrict each other; such decline can also be found in previous empirical work: for instance, economy, ecological environment, health system also faced declining coordination development due to the weakening performances of systems [70]; thus, countermeasures are urgently needed to prevent further decline.

**Fig 11:**
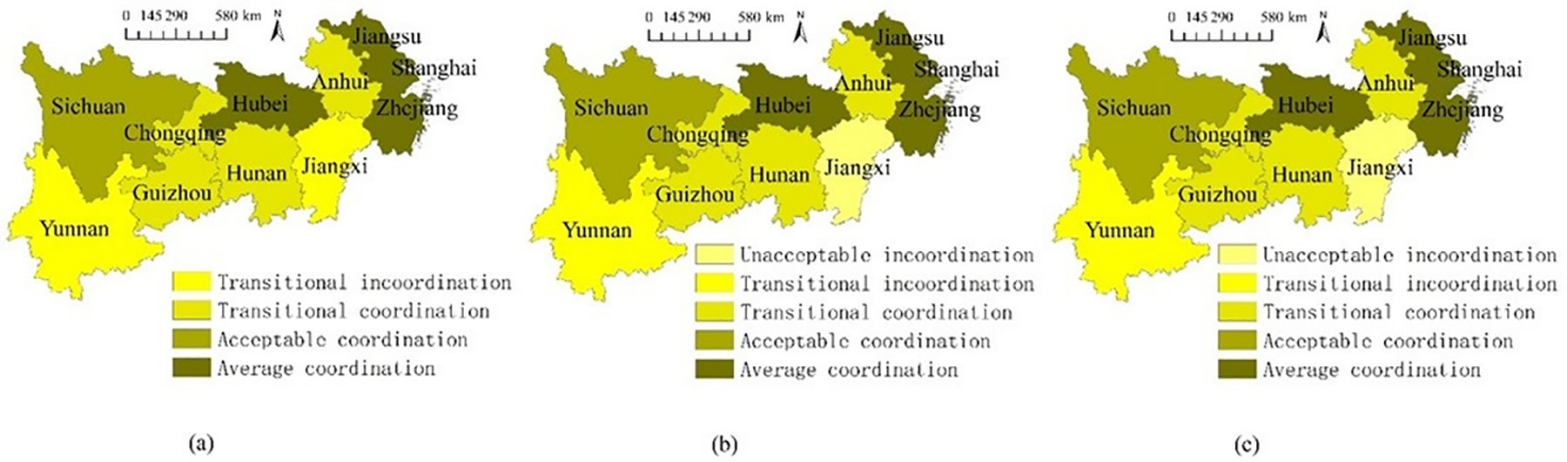
Spatial comparisons of the coordinated growth performance prediction. (a-c) 2019–2021.

The predicted spatial comparisons of the coordinated growth performances are exhibited in Fig 11. Generally, the predicted spatial variations of the coordinated growth performances will remain the same as in the past years, and the coastal regions and Hubei will still have better performances in the coordinated growth between the two systems. However, the gaps among regions (e.g., Jiangxi and the rest regions) will gradually increase. Such a different tendency proves that the benign coordinated interactions between higher education and science popularization in certain places encounter certain resistances [55, 70], so countermeasures are do needed from both the national and the local perspectives to enhance the coordinated growth between the two systems and further decrease inter-regional imbalance.

### Countermeasures and suggestions

The overall performances of the higher education system and the science popularization system vary temporally and spatially; besides, the two systems can achieve coordinated growth, and the regional differences of the coordinated growth performances between the two systems will be likely to increase, thus, considering the main influential factors affecting the coordinated growth relations between the two systems, countermeasures which are cooperative, coordinated, comprehensive, targeted, and differentiated should be proposed to accelerate regional balanced coordinated growth between the two systems. We hereby propose several countermeasures for different regions, which are also instructive to regions sharing similar conditions.

Generally, there are several suggestions for the national authority. As it has been found that in the next years, the regions with advanced coordinated growth performances will still keep better performances, whereas the regional gaps will gradually increase, implying that the national strategies aiming at enhancing high-quality and balanced development among regions will be threatened, thus, it is needed for the national authority to involve or participate in coordination activities, to reverse the differentiation trend, and to achieve regional-balanced coordinated growth between higher education and science popularization. National strategies or tactics should be implemented from the national perspective. For instance, national directional plans aiming to improve coordinated growth between higher education and science popularization can be issued so that every region can find the own suitable growth path under the general framework and thus achieve regional balance gradually; furthermore, national specialized committee can be set up to deal with inter-regional coordinated growth issues (such as higher education cooperation and science popularization assistance) by playing advisory and coordination roles so that regional balance and high-quality development can be gradually achieved.

For the regions with satisfactory overall performances and coordinated growth performances (such as Jiangsu, Shanghai, Hubei, Zhejiang, etc.), more creative approaches should be taken to maintain the coordinated growth interactions between higher education and science popularization at satisfactory status, and to help these regions become representative cases. It has been found in the study that higher education performances are spatially related to science popularization performances, and higher education performs relatively better than science popularization, implying that higher education can be effectively and wisely applied to help the coordinated growth of science popularization. In specific, these regions can effectively use higher education facilities and equipment as the carrier or resources of science popularization activities, and initiate science popularization activities with the theme of higher education development, so that the science popularization system can gradually achieve coordinated growth with higher education; besides, it is encouraged for universities to initiate volunteering sponsorship programs aiming to reduce the geographical differences of the coordinated growth; for instance, universities in the more coordinated regions can help the ones in the less coordinated regions by sending excellent university faculties or volunteers, by organizing bilateral forums, and by carrying out sponsorship programs, so that the higher education system in the lagging regions can be enhanced and thus the science popularization system can realize coordinated growth later.

For the regions at the transitioning status (0.4–0.6), the prevention of the grade decline, and the upgrade of the coordinated growth performances are the top priorities, and more resources should be devoted into both higher education and science popularization in order to enhance the coordinated growth interactions between the two systems. In this study, it has been discovered that the coordinated growth performances of most regions at the transitioning status remained stable fluctuations, implying that the coordination interactions between higher education and science popularization could keep relatively stable; therefore, it is possible for the regions to upgrade the coordination interactions based on the premise of the current status. In specific, several countermeasures can be applied; firstly, it is possible for stakeholders to maintain existing advantages, outcomes, and resources in order to prevent potential grade decline; secondly, local administration structures of both higher education and science popularization are suggested to be carefully and innovatively reformed in order to reduce obstacles of bilateral coordinated interactions; thirdly, development strategies and detailed timeline should be carefully discussed and made in order to apply higher education resources into science popularization activities and to contribute science popularization outcomes to higher education programs.

For the regions with the coordinated growth performances falling in the incoordination category, it is important to stop the decline, accelerate the coordinated growth between the two systems and reduce the gaps with other regions. In this research it has been found that several indices are playing as the main influential factors in overall performances and coordinated growth performances mainly because of their large weights, implying that these influential factors should be emphasized by these lagging regions to increase the coordinated growth: endeavoring to enhance the values of the main influential factors contribute to more efficient development of the coordinated growth between higher education and science popularization. Several suggestions may be considered for these regions; firstly, differentiated growth tactics should be considered and executed. As there are limited resources and abilities to achieve high coordinated growth performances just like the coastal areas in a short time, regions with less coordinated growth statuses such as Yunnan and Jiangxi should discover new differentiated approaches by analyzing unique strengths and opportunities, and by focusing on specific influential factors (such as the annual budget per student and the number of science and technology museums) to achieve new coordinated growth; in other words, limited resources to specific aspects; besides, it is suggested to learn from other more coordinated places and seek inter-regional or even international cooperation with these regions; by learning and cooperating, regions may understand how to focus on the key influential factors and realize high-speed coordinated growth so that spatial differences can be gradually eliminated.

## Conclusions

In this study, we firstly analyze the coordinated growth interactions between higher education and science popularization, and construct a coordination model to interpret such coordination relations. Secondly, we set up a coordination assessment system based on the coordination model, and use this system to evaluate (1) the main influential factors in the coordination mechanism, (2) the overall performances of both higher education system and science popularization system, and (3) the coordinated growth performances between the two systems. Thirdly, according to the predicted trends of the coordinated growth performances, we propose specific and applicable countermeasures to enhance the future coordinated growth performances.

The research hypotheses (H1: the overall performances of higher education and science popularization vary temporally and spatially; H2: higher education and science popularization can achieve coordinated growth with both temporal and spatial characteristics) are tested true. Besides, the temporal and spatial comparisons among regions give us new prospects to discover the coordinated growth relations between the two systems.

Some main conclusions are pointed out as follows. (1) For the main influential factors, the annual budget per student, and the number of science and technology museums affect both systems more obviously. (2) For the overall performances, the temporal fluctuations of higher education remain relatively stable, whereas the increasing tendencies of science popularization are more obvious and the gaps of the fluctuations are increasing; besides, there are similarities of the spatial distributions of the two systems, whereas the average overall performances of higher education are relatively higher than those of science popularization system. (3) For the coordinated growth performances, most regions remain mild fluctuations in the coordination or transitioning category temporally, demonstrating that the two systems keep relatively stable coordinated status; furthermore, spatially speaking, the coastal regions and Hubei perform better than other places, and Guizhou is gradually reducing the gap with other regions. (4) For the future of the coordinated growth performances, the fluctuations of most regions will be more stable with slight increase than in the past, and the coastal regions together with Hubei will still have better performances; however, the gaps between Jiangxi and the rest ones will increase. Therefore, countermeasures which are cooperative, coordinated, comprehensive, targeted, and differentiated should be considered to enhance the coordinated growth performances.

The novelties of this study are pointed out as follows. (1) We analyze the coordinated growth mechanism between higher education and science popularization, and construct the coordination model to assess their coordinated growth interactions. The coordinated growth interaction mechanism and the coordination model, which are less researched in the past literature, are beneficial for us to understand the mutual coordinated growth relations between the two systems. (2) We select representative and aggregated indices and thus construct a coordination assessment system, which changes the abstract coordination model into concrete and measurable indices; such changes are innovative because it facilitates future studies to assess the development of higher education and science popularization objectively and precisely. (3) The temporal and spatial comparisons of the overall performances and the coordinated growth performances provide more dynamic and comprehensive insights to understand the coordinated growth relations between the two systems, which contributes to existing research in the fields of higher education and science popularization; besides, the temporal and spatial predictions of the coordinated growth performances, and the suggestions based on the predictions provide new lights to take proactive countermeasures in order to enhance the coordination growth between the two systems.

Of course, we must admit some limitations of this study. (1) Only 9 years’ data are collected due to data accessibility, and if possible, more data should be collected for more detailed analysis. (2) Some indices, such as the direct fiscal contributions from higher education institutes to science popularization activities, are neglected due to data availability. We may endeavor to find these data in the future studies so that more comprehensive and detailed explorations of the coordinated growth between the two systems can be realized.

## Supporting information

S1 Table Overall performance of the higher education system. (DOCX)

S2 Table Overall performance of the science popularization system. (DOCX)

S3 Table Coordinated growth performance between two systems. (DOCX)

S4 Table Predictions of the coordinated growth performance. (DOCX)
