# Supplementary material for: Higher education and science popularization: Can they achieve coordinated growth?
Source: PLoS One. 2021 Sep 7;16(9):e0256612. doi: 10.1371/journal.pone.0256612 (PMC8423295; doi:10.1371/journal.pone.0256612)
Supplement: S1 Table — (DOCX) [file pone.0256612.s001.docx]

**S1 Table. Overall Performance of the Higher Education System.**

|  | **2010** | **2011** | **2012** | **2013** | **2014** | **2015** | **2016** | **2017** | **2018** | **Mean** | **Average OP** |
| --- | --- | --- | --- | --- | --- | --- | --- | --- | --- | --- | --- |
| **Shanghai** | 0.660 | 0.658 | 0.643 | 0.610 | 0.615 | 0.581 | 0.569 | 0.570 | 0.606 | 0.612 | Fair |
| **Jiangsu** | 0.641 | 0.607 | 0.617 | 0.628 | 0.660 | 0.681 | 0.703 | 0.697 | 0.652 | 0.654 | Fair |
| **Zhejiang** | 0.437 | 0.397 | 0.408 | 0.397 | 0.422 | 0.416 | 0.429 | 0.439 | 0.433 | 0.420 | Average |
| **Anhui** | 0.356 | 0.360 | 0.379 | 0.343 | 0.392 | 0.358 | 0.371 | 0.345 | 0.336 | 0.360 | Acceptable |
| **Jiangxi** | 0.286 | 0.280 | 0.354 | 0.272 | 0.293 | 0.280 | 0.258 | 0.258 | 0.267 | 0.283 | Acceptable |
| **Hubei** | 0.484 | 0.492 | 0.485 | 0.499 | 0.491 | 0.487 | 0.508 | 0.499 | 0.481 | 0.492 | Average |
| **Hunan** | 0.392 | 0.409 | 0.422 | 0.382 | 0.399 | 0.362 | 0.381 | 0.390 | 0.389 | 0.392 | Acceptable |
| **Chongqing** | 0.301 | 0.324 | 0.325 | 0.265 | 0.303 | 0.280 | 0.307 | 0.292 | 0.271 | 0.296 | Acceptable |
| **Sichuan** | 0.476 | 0.445 | 0.482 | 0.427 | 0.477 | 0.478 | 0.473 | 0.485 | 0.508 | 0.472 | Average |
| **Guizhou** | 0.169 | 0.055 | 0.000 | 0.134 | 0.167 | 0.171 | 0.118 | 0.213 | 0.238 | 0.141 | Unacceptable |
| **Yunnan** | 0.268 | 0.238 | 0.239 | 0.203 | 0.232 | 0.254 | 0.258 | 0.159 | 0.198 | 0.228 | Acceptable |
