# Supplementary material for: Higher education and science popularization: Can they achieve coordinated growth?
Source: PLoS One. 2021 Sep 7;16(9):e0256612. doi: 10.1371/journal.pone.0256612 (PMC8423295; doi:10.1371/journal.pone.0256612)
Supplement: S2 Table — (DOCX) [file pone.0256612.s002.docx]

**S2 Table. Overall Performance of the Science Popularization System.**

|  | **2010** | **2011** | **2012** | **2013** | **2014** | **2015** | **2016** | **2017** | **2018** | **Mean** | **Average OP** |
| --- | --- | --- | --- | --- | --- | --- | --- | --- | --- | --- | --- |
| **Shanghai** | 0.583 | 0.594 | 0.600 | 0.600 | 0.634 | 0.653 | 0.670 | 0.680 | 0.697 | 0.635 | Fair |
| **Jiangsu** | 0.401 | 0.397 | 0.424 | 0.455 | 0.455 | 0.396 | 0.456 | 0.483 | 0.511 | 0.442 | Average |
| **Zhejiang** | 0.419 | 0.386 | 0.397 | 0.478 | 0.443 | 0.470 | 0.470 | 0.563 | 0.548 | 0.464 | Average |
| **Anhui** | 0.304 | 0.355 | 0.323 | 0.350 | 0.345 | 0.332 | 0.237 | 0.390 | 0.409 | 0.338 | Acceptable |
| **Jiangxi** | 0.240 | 0.204 | 0.232 | 0.265 | 0.217 | 0.142 | 0.206 | 0.131 | 0.091 | 0.192 | Unacceptable |
| **Hubei** | 0.607 | 0.607 | 0.581 | 0.552 | 0.558 | 0.550 | 0.509 | 0.600 | 0.560 | 0.569 | Average |
| **Hunan** | 0.189 | 0.264 | 0.306 | 0.229 | 0.282 | 0.227 | 0.302 | 0.283 | 0.304 | 0.265 | Acceptable |
| **Chongqing** | 0.252 | 0.250 | 0.267 | 0.260 | 0.317 | 0.332 | 0.337 | 0.342 | 0.357 | 0.301 | Acceptable |
| **Sichuan** | 0.342 | 0.337 | 0.399 | 0.336 | 0.335 | 0.288 | 0.344 | 0.381 | 0.426 | 0.354 | Acceptable |
| **Guizhou** | 0.112 | 0.091 | 0.171 | 0.175 | 0.151 | 0.157 | 0.182 | 0.176 | 0.228 | 0.160 | Unacceptable |
| **Yunnan** | 0.260 | 0.271 | 0.231 | 0.265 | 0.260 | 0.250 | 0.300 | 0.324 | 0.302 | 0.274 | Acceptable |
