# Supplementary material for: Higher education and science popularization: Can they achieve coordinated growth?
Source: PLoS One. 2021 Sep 7;16(9):e0256612. doi: 10.1371/journal.pone.0256612 (PMC8423295; doi:10.1371/journal.pone.0256612)
Supplement: S3 Table — (DOCX) [file pone.0256612.s003.docx]

**S3 Table. Coordinated Growth Performance between Two Systems.**

|  | **2010** | **2011** | **2012** | **2013** | **2014** | **2015** | **2016** | **2017** | **2018** |
| --- | --- | --- | --- | --- | --- | --- | --- | --- | --- |
| **Shanghai** | 0.788 | 0.791 | 0.788 | 0.778 | 0.790 | 0.785 | 0.786 | 0.789 | 0.806 |
| **Jiangsu** | 0.712 | 0.701 | 0.715 | 0.731 | 0.740 | 0.720 | 0.752 | 0.762 | 0.760 |
| **Zhejiang** | 0.654 | 0.625 | 0.635 | 0.660 | 0.658 | 0.665 | 0.670 | 0.705 | 0.698 |
| **Anhui** | 0.574 | 0.598 | 0.592 | 0.589 | 0.607 | 0.587 | 0.544 | 0.606 | 0.609 |
| **Jiangxi** | 0.512 | 0.489 | 0.535 | 0.518 | 0.502 | 0.446 | 0.480 | 0.429 | 0.395 |
| **Hubei** | 0.736 | 0.739 | 0.729 | 0.725 | 0.724 | 0.719 | 0.713 | 0.740 | 0.721 |
| **Hunan** | 0.522 | 0.573 | 0.599 | 0.544 | 0.579 | 0.536 | 0.582 | 0.576 | 0.587 |
| **Chongqing** | 0.525 | 0.533 | 0.542 | 0.512 | 0.557 | 0.552 | 0.567 | 0.562 | 0.558 |
| **Sichuan** | 0.635 | 0.622 | 0.662 | 0.616 | 0.632 | 0.609 | 0.635 | 0.656 | 0.682 |
| **Guizhou** | 0.371 | 0.266 | 0.000 | 0.391 | 0.399 | 0.404 | 0.383 | 0.440 | 0.482 |
| **Yunnan** | 0.514 | 0.504 | 0.484 | 0.481 | 0.496 | 0.502 | 0.527 | 0.476 | 0.494 |
