# Supplementary material for: Higher education and science popularization: Can they achieve coordinated growth?
Source: PLoS One. 2021 Sep 7;16(9):e0256612. doi: 10.1371/journal.pone.0256612 (PMC8423295; doi:10.1371/journal.pone.0256612)
Supplement: S4 Table — (DOCX) [file pone.0256612.s004.docx]

**S4 Table. Predictions of the Coordinated Growth Performance.**

|  | ***a*** | ***P*** | ***r*** | **Year of Prediction** | | |
| --- | --- | --- | --- | --- | --- | --- |
|  |  |  |  | **2019** | **2020** | **2021** |
| **Shanghai** | -0.002 | 0.556 | 1.533 | 0.796 | 0.798 | 0.799 |
| **Jiangsu** | -0.011 | 0.889 | -0.123 | 0.773 | 0.781 | 0.790 |
| **Zhejiang** | -0.016 | 1.000 | 1.175 | 0.714 | 0.725 | 0.737 |
| **Anhui** | 0.000 | 0.556 | 1.347 | 0.591 | 0.591 | 0.591 |
| **Jiangxi** | 0.033 | 0.889 | 0.333 | 0.407 | 0.394 | 0.381 |
| **Hubei** | 0.002 | 0.667 | 1.573 | 0.720 | 0.719 | 0.717 |
| **Hunan** | -0.001 | 0.667 | 0.934 | 0.575 | 0.576 | 0.576 |
| **Chongqing** | -0.009 | 0.889 | 0.590 | 0.571 | 0.577 | 0.582 |
| **Sichuan** | -0.008 | 0.667 | 0.820 | 0.663 | 0.668 | 0.673 |
| **Guizhou** | -0.065 | 0.667 | 1.013 | 0.507 | 0.541 | 0.578 |
| **Yunnan** | -0.001 | 0.667 | 0.938 | 0.498 | 0.498 | 0.499 |
